# *Babesia vesperuginis* in insectivorous bats from China

**DOI:** 10.1186/s13071-018-2902-9

**Published:** 2018-05-29

**Authors:** Hui-Ju Han, Jian-Wei Liu, Hong-Ling Wen, Xiang-Rong Qin, Min Zhao, Li-Jun Wang, Chuan-Min Zhou, Rui Qi, Hao Yu, Xue-Jie Yu

**Affiliations:** 1 Wuhan University School of Health Sciences, Wuhan, Hubei China; 2 Shandong University School of Public Health, Jinan, Shandong China; 3 Fudan University School of Medicine, Shanghai, China

**Keywords:** Bat, China, *Babesia vesperuginis*

## Abstract

**Background:**

To increase understanding of human bacterial and parasitic pathogens in bats, we investigated the prevalence of *Babesia* spp., *Rickettsia* spp., *Anaplasma* spp. and *Coxiella burnetii* in bats from China.

**Methods:**

Bats were captured from Mengyin County, Shandong Province of China using nets. DNA was extracted from the blood and spleen of bats for molecular detection of *Babesia* spp., *Rickettsia* spp., *Anaplasma* spp. and *Coxiella burnetii* with specific primers for each species.

**Results:**

A total of 146 spleen samples and 107 blood samples of insectivorous bats, which belonged to 6 species within two families, were collected from Mengyin County, Shandong Province of China. We found that two *Eptesicus serotinus* (2/15, 13.3%) were positive for *Babesia vesperuginis*. We were unable to detect genomic sequences for *Rickettsia* spp., *Anaplasma* spp. and *Coxiella burnetii*.

**Conclusions:**

To our knowledge, our study showed for the first time the presence of *Babesia vesperuginis* in *Eptesicus serotinus* collected from China, suggesting that *Babesia vesperuginis* has a broad host species and geographical distribution.

## Background

Bats have been studied in recent years due to their association with several serious emerging viruses, such as SARS-Coronavirus, Hendra virus, Nipha virus, Ebola virus and Marburg virus [1]. Most studies have focused on emerging viruses; however, bacterial and parasitic agents in bats have been largely neglected. We previously showed that bats from northern China carried several novel *Bartonella* spp. [2] as well as a diversity of pathogenic *Leptospira* spp. [3]. To have a better understanding of bacterial and parasitic pathogens in bats, we expanded our study to several tick-borne bacterial and parasitic pathogens, including *Babesia* spp., *Rickettsia* spp., *Anaplasma* spp. and *Coxiella burnetii*.

*Babesia* spp. are tick-transmitted protozoan hemoparasites associated with a wide range of vertebrate hosts worldwide [4]. So far, *Babesia* spp. detected in bats have been almost exclusively *Babesia vesperuginis* [5–9], with the exception of a study reporting *Babesia canis*, the causative agent of canine babesiosis, in the feces of bats from Hungary [10]. In addition, a recent study reported the detection of *B*. *vesperuginis*, *Babesia crassa* and *B*. *canis* in ixodid ticks on bats [11], which indicated that bats could harbor a greater diversity of *Babesia* species and hard ticks could also play a role in *Babesia* transmission among bats. The role of bats in the ecology of *Babesia* spp. as well as the vectors involved in transmission of *Babesia* spp. among bats deserves further investigation.

*Rickettsia* spp. are intracellular bacteria that are responsible for life-threatening spotted and typhus fevers in humans [12]. So far, *Rickettsia* spp. infections in bats were limited to several serological and molecular surveys in America, Africa and Europe. Antibodies against several spotted fever group (SFG) *Rickettsia* spp. were reported in bats from Brazil and USA [13, 14]. DNA of *Rickettsia* spp. was also detected in the blood samples of bats from Swaziland, South Africa and Saint Kitts Island [15, 16]. A recent study conducted in Europe showed that *Rickettsia* spp. DNA was detected in bat feces [17]. Moreover, bat ectoparasites, including soft ticks (Argasidae), hard ticks (Ixodidae) and flies (Nycteribiidae), were found to carry a diversity of *Rickettsia* spp. that were identical to those found in bats, indicating the vector-borne transmission of *Rickettsia* spp. [18–23]. So far, there is a lack of knowledge on *Rickettsia* spp. in bats in Asia.

*Anaplasma* spp. belong to the order Rickettsiales, causing tick-borne anaplasmosis in animals and humans [24]. So far, there is no report of *Anaplasma* spp. in bats.

*Coxiella burnetii* is an obligate intracellular gram-negative bacterium, and is the agent of Q fever [25]. So far, there are no reports of *C*. *burnetii* in bats. However, its existence in ticks from bats has been reported in Algeria [26].

Therefore, the aim of the study was to investigate the prevalence of *Babesia* spp., *Rickettsia* spp., *Anaplasma* spp. and *C*. *burnetii* in bats from China.

## Methods

### Bat sampling

Bats were captured with nets from Mengyin County, Shandong Province of China (117°45' to 118°15'N,35°27' to 36°02'S) as part of an ongoing program of detecting novel microorganisms (viruses, bacteria and parasites) in bats. Identification of bat species was performed by DNA sequencing the PCR amplified cytochrome *b* (*cytb*) gene as described previously [27]. Details on the collection of bat specimens are as described previously [2]. Briefly, bats were anesthetized for collecting blood samples, and were then killed with overdosed anesthetic to collect organs.

### Molecular detection for *Babesia* spp., *Rickettsia* spp., *Anaplasma* spp. and *C*. *burnetii*

Bat blood DNA extraction was performed with the Qiagen DNA Kit (Qiagen, Hilden, Germany) and the spleen was extracted with the AllPrep DNA/RNA Mini Kit (Qiagen), according to the manufacturer’s instructions. Blood DNA samples were screened for *Babesia* spp., *Rickettsia* spp. and *Anaplasma* spp. Spleen DNA samples were screened for *C*. *burnetii*. Primers used in this study are shown in Table 1.

**Table 1:** PCR primers used for *Babesia* spp., *Rickettsia* spp. and *Anaplasma* spp. and *C*. *burnetii* screening

Target agent	PCR method	Primer	Primer sequences (5'→3')	Target gene	Amplicon size (bp)	Tissue tested	Reference
*Babesia* spp.	PCR	BJ1	GTCTTGTAATTGGAATGATGG	*18S* rDNA	~500	Blood	[10]
		BN2	TAGTTTATGGTTAGGACTACG				
	Nested PCR	Bab_For1	ATWGGATTYTATATGAGTAT	*cox*1	924		[7]
		Bab_Rev1	ATAATCWGGWATYCTCCTTGG				
		Bab_For2	TCTCTWCATGGWTTAATTATGATAT				
		Bab_Rev2	TAGCTCCAATTGAHARWACAAAGTG				
*Rickettsia* spp.	qPCR	gltA-F	GTGAATGAAAGATTACACTATTTAT	*gltA*	–	Blood	[30]
		gltA-R	GTATCTTAGCAATCATTCTAATAGC				
	qPCR	338-F	GAMAAATGAATTATATACGCCGCAAA	RC0338 gene	–		
		338-R	ATTATTKCCAAATATTCGTCCTGTAC				
*Anaplasma* spp.	Nested PCR	AE1-F	AAGCTTAACACATGCAAGTCGAA	*16S* rRNA	926	Blood	[31]
		AE1-R	AGTCACTGACCCAACCTTAAATG				
		EE3	GTCGAACGGATTATTCTTTATAGCTTGC				
		EE4	CCCTTCCGTTAAGAAGGATCTAATCTCC				
*Coxiella burnetii*	Nested PCR	omp1	AGTAGAAGCATCCCAAGCATTG	*com*1	438	Spleen	[32]
		omp2	TGCCTGCTAGCTGTAACGATTG				
		omp3	GAAGCGCAACAAGAAGAACA				
		omp4	TGGAAGTTATCACGCAGTTG				

For *Babeisa* spp., an initial screening PCR targeting *18S* rDNA was conducted in a 50 μl mixture containing 25 μl DreamTaq Green PCR Master Mix (2×) (Thermo Fisher Scientific, Waltham, MA, USA), 0.8 μl 25 μmol/l of each forward and reverse primer (Sangon Biotech, Shanghai, China), 16.4 μl nuclease-free water, and 7 μl blood DNA of each sample. Nuclease-free water was used as negative controls. PCR was performed under the following conditions: 1 denaturing cycle at 95 °C for 5 min followed by 35 cycles at 95 °C for 30 s, 55°C for 30 s, and 72 °C for 1 min and an additional final cycle at 72 °C for 10 min.

For *18S* rDNA positive samples, an additional nested PCR targeting *cox*1was performed. The first round PCR was conducted in a 25 μl mixture containing 0.125 μl 5 U/μl TakaRa Ex Taq (TaKaRa, Shiga, Japan), 2.5 μl 10×ExTaqbuffer (Mg^2+^ free), 2 μl 25 mM MgCl_2_, 2 μl dNTP mixture (2.5 mM for each), 0.4 μl 25 μmol/l of each forward and reverse primer, 12.6μl nuclease-free water and 5 μl blood DNA of each sample. The second round PCR was the same as described above for *18S* rDNA except that 3 μl of first round PCR product was used as a template. The PCR condition was the same as described for *18S* rDNA, but the annealing temperature for the first and second rounds of PCR were 45 °C and 49 °C, respectively.

For *Anaplasma* spp. and *C*. *burnetii*, a nested PCR was conducted as described for *cox*1 of *Babesia* spp. Blood DNA and spleen DNA were used for the detection of *Anaplasma* spp. and *C*. *burnetii*, respectively. The PCR conditions were the same as described for *18S* rDNA of *Babesia* spp.

PCR products were analyzed by 1.2% agarose gel electrophoresis and detected using ethidium bromide under UV light. PCR products with expected sizes were excised from gels and extracted using a Gel Extraction Kit (Promega, Madison, WI, USA), which were then cloned into the pMD19-T vector (TaKaRa) for sequencing.

Quantitative real-time PCR (qPCR) was used for the detection of *Rickettsia* spp. The reaction was conducted in a 50 μl mixture containing 25 μl FastStart Universal SYBR Green Master (ROX), 0.8 μl 25 μmol/l of each forward and reverse primer, 16.4 μl nuclease-free water, and 7 μl blood DNA of each sample. The tests were performed using a Light Cycler 480 II (Roche, Mannheim, Germany) with the following conditions: an initial denaturation at 95 °C for 10 min, followed by 40 cycles at 95 °C for 10 s and at 58 °C for 30 s. Nuclease-free water was used as negative controls in each run. Results were considered positive if the cycle threshold (Ct) value was lower than 36.

### Phylogenetic analysis

Chromatograms were checked with Chromas 2.5.1 (Technelysium, Tewantin, QLD, Australia) to exclude double peaks, and sequences were analyzed with the BLAST programme (http://blast.ncbi.nlm.nih.gov/Blast.cgi). After alignment by ClustalW with MEGA 7.0 [28], phylogenetic trees were constructed using the Maximum Likelihood method with the Tamura-Nei model by using MEGA7.0, and bootstrap values were calculated with 1000 replicates.

## Results

A total of 146 bats belonging to 6 species within two families were sampled. Bats of the family Rhinolophidae included 4 *Rhinolophus ferrumequinum* and 14 *Rhinolophus pusillus* captured from a karst cave; bats of the family of Vespertilionidae included 26 *Eptesicus serotinus* from two farmers’ houses, 34 *Myotis fimbriatus* and 10 *Myotis ricketti* from a city sewer and 58 *Myotis pequinius* from a cave (Table 2). Finally, 146 spleen DNA samples were screened for *C*. *burnetii*, and 107 blood DNA samples were screened for *Babesia* spp., *Rickettsia* spp. and *Anaplasma* spp.

**Table 2:** Information of bats sampled from Mengyin County, Shandong Province of China

Family	Sampling site	Species	Common name	Spleen samples	Blood samples
Rhinolophidae	Karst Cave	*Rhinolophus ferrumequinum*	Greater horseshoe bat	4	3
		*Rhinolo phuspusillus*	Least horseshoe bat	14	10
Vespertilionidae	Farmers’ houses	*Eptesicus serotinus*	Common serotine	26	15
	City sewer	*Myotis fimbriatus*	Fringed long-footed myotis	34	16
		*Myotis ricketti*	Rickett’s big-footed myotis	10	5
	Cave	*Myotis pequinius*	Peking myotis	58	58
Total				146	107

**Fig. 1:**
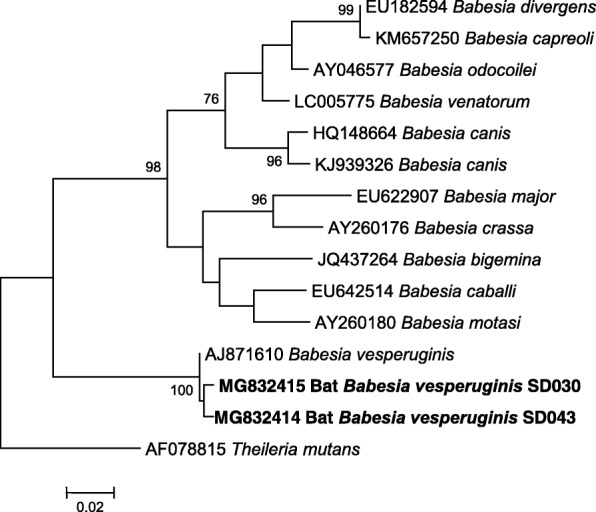
Phylogenetic tree based on the 517 bp *18S* rDNA sequences of *Babesia* spp. identified in this study and relevant sequences from GenBank. The tree was constructed with MEGA 7.0 by using the Maximum Likelihood method with the Tamura-Nei model. Only bootstrap values no lower than 75% were shown. *Babesia vesperuginis* detected in bats in this study are shown in bold, and are designated as bat *Babesia vesperuginis* SD030 and bat *Babesia vesperuginis* SD043. *Theileria mutans* was used as the outgroup

**Fig. 2:**
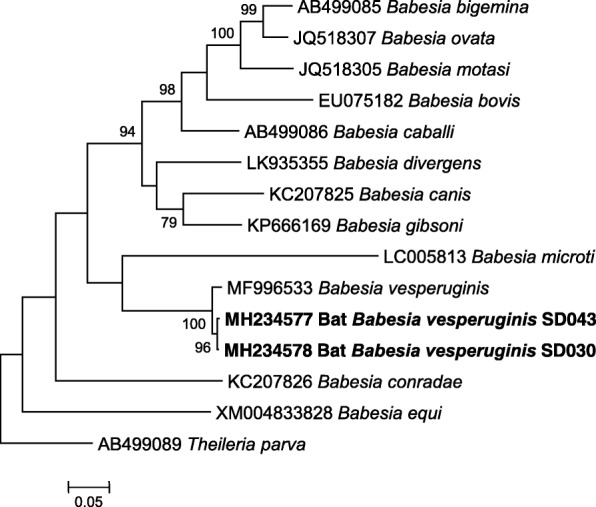
Phylogenetic tree based on the 924 bp *cox*1 sequences of *Babesia* spp. identified in this study and relevant sequences from the GenBank. The tree was constructed with MEGA 7.0 by using the Maximum Likelihood method with the Tamura-Nei model. Only bootstrap values no lower than 75% were shown. *Babesia vesperuginis* detected in bats in this study are shown in bold, and are designated as bat *Babesia vesperuginis* SD030 and bat *Babesia vesperuginis* SD043. *Theileria parva* was used as the outgroup

In this study, we found that 2 out of 15 blood samples of *E*. *serotinus* (2/15, 13.3%) were positive for *Babesia* spp., while blood samples of the other 5 bat species (*Rh*. *ferrumequinum*, *Rh*. *pusillus*, *My*. *fimbriatus*, *My*. *ricketti* and *My*. *pequiniu*) were all negative. BLAST analysis of the 517 bp *18S* rDNA sequences showed that the two *Babesia* spp. detected in *E*. *serotinus* in this study (designated as bat *Babesia vesperuginis* SD030 and bat *Babesia vesperuginis* SD043), which differed by 4 nucleotides, shared 99.4% similarity with *B*. *vesperuginis* (GenBank: AJ871610). BLAST analysis of the 924 bp *cox*1 sequences showed that the bat *Babesia vesperuginis* SD030 and bat *Babesia vesperuginis* SD043 differed by 3 nucleotides, and shared 98.2% and 98.1% similarity with *B*. *vesperuginis* (GenBank: MF996533), respectively. Phylogenetic analysis of *18S* rDNA and *cox*1genes also showed that *Babeisa* spp. detected in bats in this study clustered together with *B*. *vesperuginis* (Figs. 1 and 2). The *18S* rDNA and *cox*1 sequences of *B*.*vesperuginis* of this study were deposited in the GenBank with accession numbers: MG832414-MG832415 and MH234577-MH234578.

We were unable to detect genomic sequences for *Rickettsia* spp., *Anaplasma* spp. and *C*. *burnetii*.

## Discussion

*Babesia vesperuginis* in bats was first described in bats from Italy, and later also found in bats from other parts of Europe (UK, Austria, Czech Republic, Romania) and South America (Colombia) [5, 7–9]. So far, *B*. *vesperuginis* has been detected in *Nyctalus noctula* and *Pipistrellus* sp. from Italy; *My*. *mystacinus* and *Pipistrellus* sp. from the UK; *Mormoops megalophylla* from Colombia; *My*. *alcathoe*, *My*. *bechsteinii*, *My*. *myotis* and *Vespertilio murinus* from Romania; *Ny*. *noctula*, *Pi*. *nathusii* and *Pi*. *pipistrellus* from the Czech Republic; *Pi*. *pipistrellus* and *Ve*. *murinus* from Austria; and *Pi*. *pipistrellus* from China [5–9]. The prevalence of *B*. *vesperuginis* in *Pipistrellus* spp. in Europe has been reported as 8.45% (6/71), 9.22% (19/206), 16.7% (6/36) and 10% (5/48) [6, 7, 9, 29]. The prevalence of *B*. *vesperuginis* in *Mo*. *megalophylla* in South America and in *N*. *noctula* in Europe was reported to be 1.19% (2/168) and 1.63% (4/246), respectively [5, 7]. However, the prevalence of *B*. *vesperuginis* in other bat species might be biased due to the limited sample size [7, 8]. In this study, the prevalence of *B*. *vesperuginis* in *E*. *serotinus* from China was 13.3% (2/15), which might also be biased by the limited sample size. To our knowledge, this is the first report of *B*. *vesperuginis* in *E*. *serotinus*, suggesting that *B*. *vesperuginis* has a broad host species and geographical distribution.

Natural and experimental infection showed that *B*.*vesperuginis* was pathogenic to bats, which could result in symptoms such as lowered blood haemoglobin, raised white blood cell counts and enlarged spleen in bats [8]. Soft ticks (*Argas vespertilionis*) were suspected to play a role in the transmission of *B*. *vesperuginis* among bats [8]. Although no ticks were found on bats in this study, a recent study reported that soft ticks (*Argas vespertilionis*) collected from *B*. *vesperuginis-*positive bats (*Pi*. *pipistrellus*) were also positive for *B*. *vesperuginis* in northwestern China [6], indicating that soft ticks might be the vector for *B*. *vesperuginis* transmission among bats.

## Conclusions

We detected *B*. *vesperuginis* in *E*. *serotinus* collected from China, suggesting that *B*. *vesperuginis* has a broad host species and geographical distribution. Since *B*. *vesperuginis* is pathogenic to bats, the finding of this species in China has some implications for the conservation of bats in China.
